# Supplementary material for: Myeloblasts transition to megakaryoblastic immunophenotypes over time in some patients with myelodysplastic syndromes
Source: PLoS One. 2023 Sep 20;18(9):e0291662. doi: 10.1371/journal.pone.0291662 (PMC10511088; doi:10.1371/journal.pone.0291662)
Supplement: S1 Fig — Lane A: Cells with low SSC were separated on the CD34 vs. CD45 plot, and CD34+ blasts (red dots), CD34-dull or -negative myeloid cells, and lymphocytes were gated. Lane B: Data from cells stained with 6-color. CD34+ blasts (red dots) are positive for CD41 expression. Lane C (negative control 1): Data from cells stained with CD33-PE, CD45-PerCP, and CD34-APC. Lane D (negative control 2): Data from cells stained with CD45-PerCP and CD34-APC. Lane E: Data from cells stained with 6-color. The CD34+CD41+ cells were negative for both GPA and CD16. (DOCX) [file pone.0291662.s001.docx]

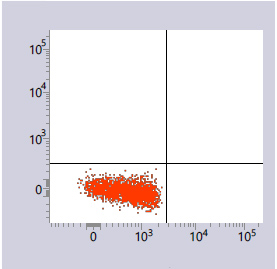

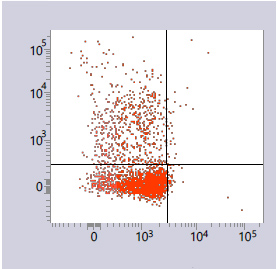

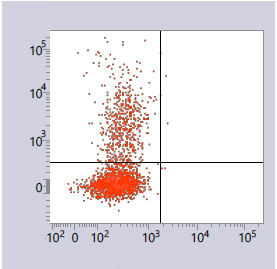

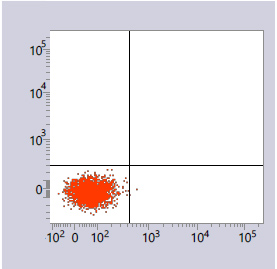

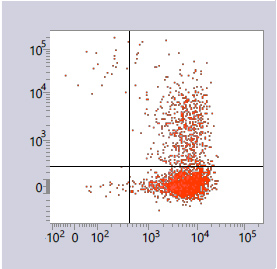

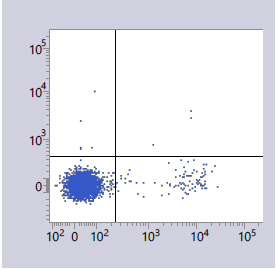

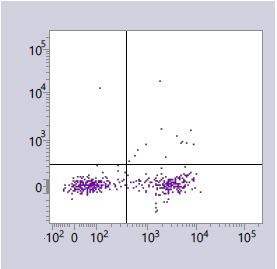

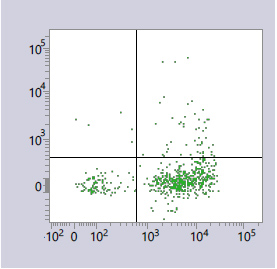

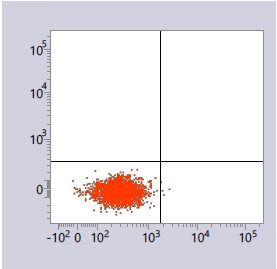

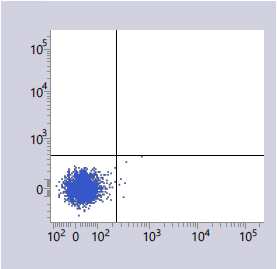

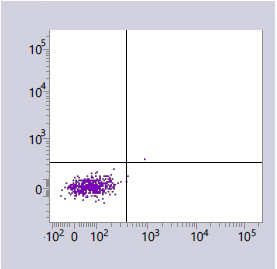

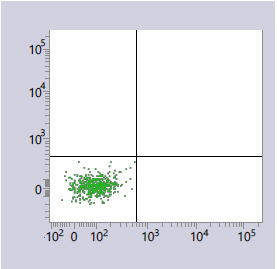

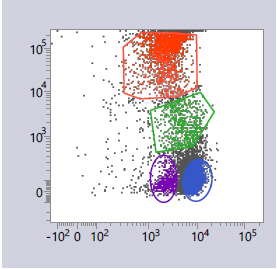

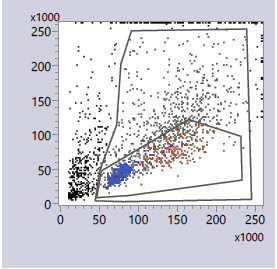

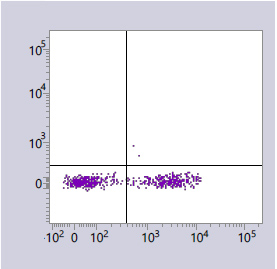

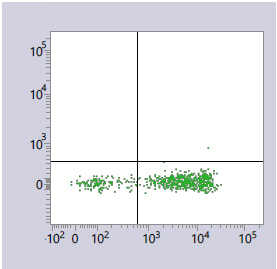

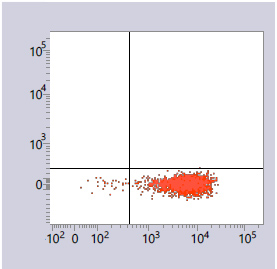

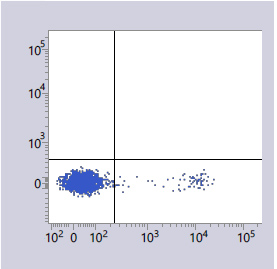


**Supplementary Figure 1 (Case 1)**

**Case 1**

Red dots: CD34+ blasts

Green dots: CD34-dull myeloid cells

Purple dots: CD34-negative myeloid cells

Blue dots: Lymphocytes

CD41-PE-Cy7

CD41-PE-Cy7

-PE-Cy87

CD34-APC

FSC

CD45-PerCP-Cy5.5

APC-Cy7 (unstained)

PE-Cy7(unstained)

CD41-PE-Cy7

CD16-APC-Cy7

GPA-FITC

**E**

**D**

**C**

**B**

**A**

FITC (unstained)

PE-Cy7 (unstained)

SSC

PE-Cy7 (unstained)

-PE-Cy87

PE (unstained)

CD33-PE

CD33-PE


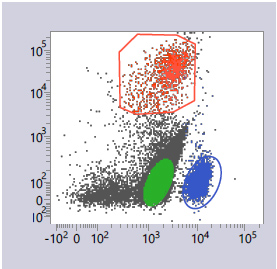

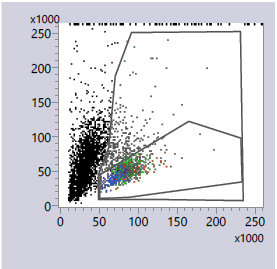


**Case 2**

Red dots: CD34+ blasts

Green dots: CD34-negative myeloid cells

Blue dots: Lymphocytes

CD16-APC-Cy7

GPA-FITC

FSC

CD45-PerCP-Cy5.5

**E**

**D**

**C**

**B**

**A**

SSC

CD34-APC

PE-Cy7 (unstained)

-PE-Cy87


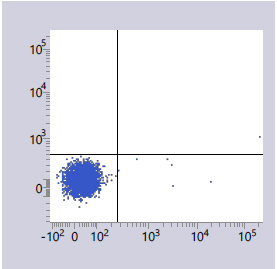

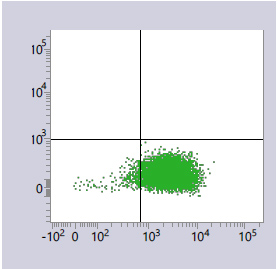

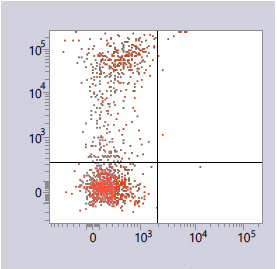

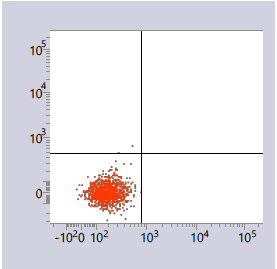

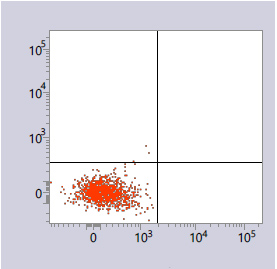

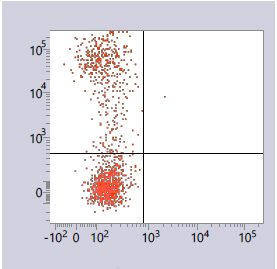

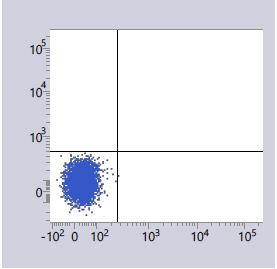

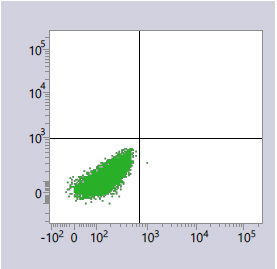

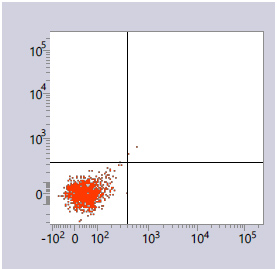

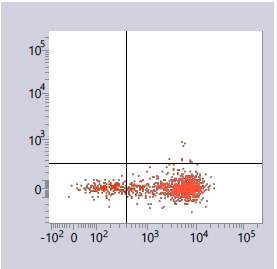

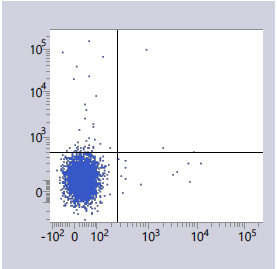

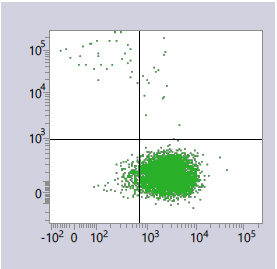

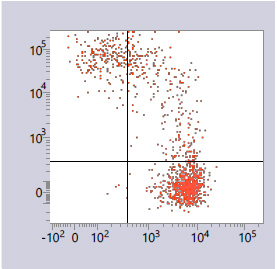


CD41-PE-Cy7

PE (unstained)

CD33-PE

CD33-PE

CD41-PE-Cy7

APC-Cy7 (unstained)

FITC (unstained)

PE-Cy7 (unstained)

PE-Cy7 (unstained)

CD41-PE-Cy7

-PE-Cy87

**Supplementary Figure 1 (Case 2)**
