# Supplementary figures and images for: Myeloblasts transition to megakaryoblastic immunophenotypes over time in some patients with myelodysplastic syndromes
Source: PLoS One. 2023 Sep 20;18(9):e0291662. doi: 10.1371/journal.pone.0291662 (PMC10511088; doi:10.1371/journal.pone.0291662)

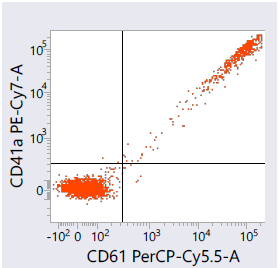
　
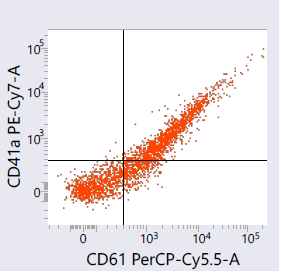


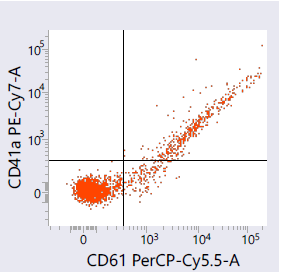
　
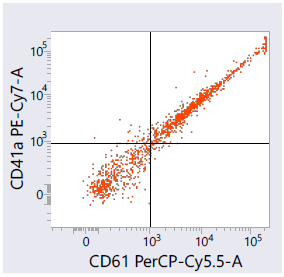


**Supplementary Figure 2.**

Supplement: S2 Fig — The data from four patients are presented. The data of one remaining case are shown in Fig 3. (DOCX) [file pone.0291662.s002.docx]
