# Supplementary material for: Myeloblasts transition to megakaryoblastic immunophenotypes over time in some patients with myelodysplastic syndromes
Source: PLoS One. 2023 Sep 20;18(9):e0291662. doi: 10.1371/journal.pone.0291662 (PMC10511088; doi:10.1371/journal.pone.0291662)
Supplement: S3 Fig — The data from three cases (Cases 2, 3, and 5 in Table 3) are shown. Panels A and B in each case show data when CD34+ blasts (red dots) were negative for CD41. The panels C-F in each case are data when CD34+ blasts (red dots) became positive for CD41 with time (D). Other cell fractions (green and blue dots) were negative for CD41 (E and F). (DOCX) [file pone.0291662.s003.docx]

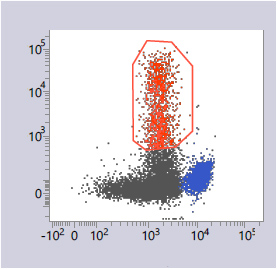


CD34-APC

CD45-PerCP-Cy5.5

**(A)**


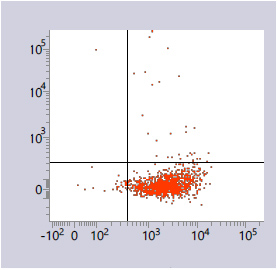


CD41-PE-Cy7

CD33-PE

**(B)**


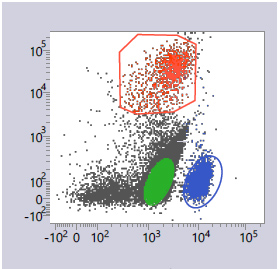


CD34-APC

CD45-PerCP-Cy5.5

**(C)**


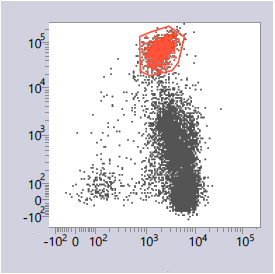


CD34-APC

CD45-PerCP-Cy5.5

**(A)**


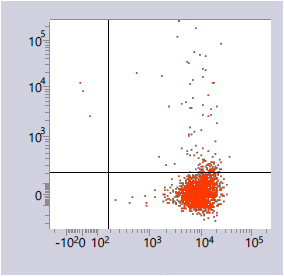


CD41-PE-Cy7

CD33-PE

**(B)**


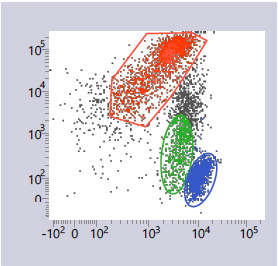


CD34-APC

CD45-PerCP-Cy5.5

**(C)**


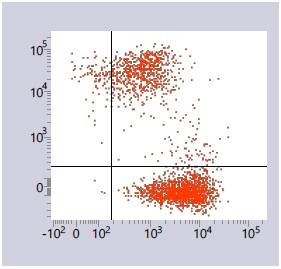

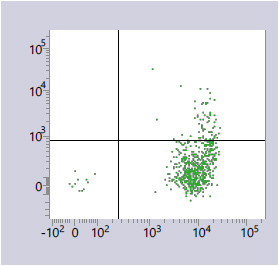

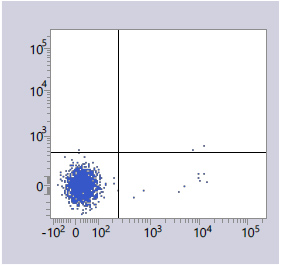


CD33-PE

CD41-PE-Cy7

-PE-Cy87

**(D)**

**(E)**

**(F)**


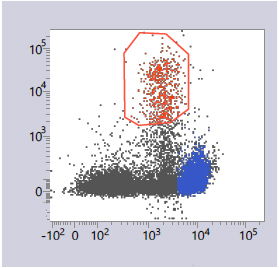


CD34-APC

CD45-PerCP-Cy5.5

**(A)**


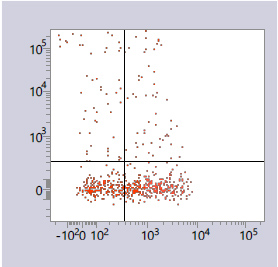


CD41-PE-Cy7

CD33-PE

**(B)**


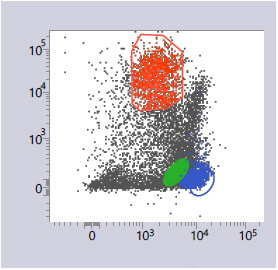


CD34-APC

CD45-PerCP-Cy5.5

**(C)**


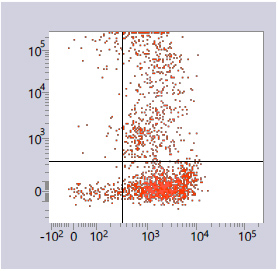

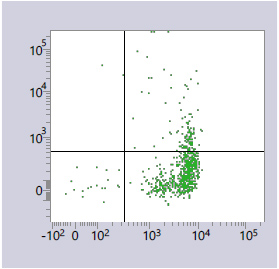

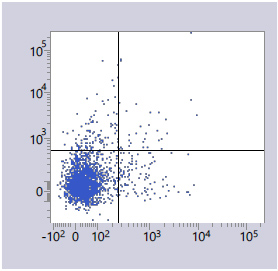


CD33-PE

CD41-PE-Cy7

-PE-Cy87

**(D)**

**(E)**

**(F)**

CD41-PE-Cy7

-PE-Cy87


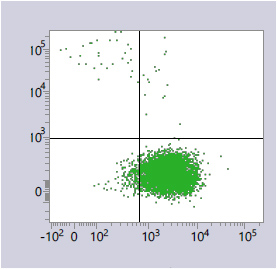


**(E)**


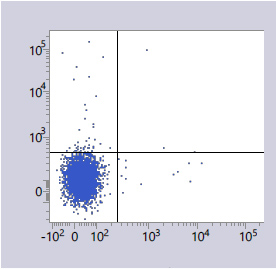


**(F)**


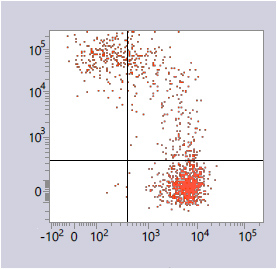


**(D)**

CD33-PE

**Supplementary Figure 3.**

**Case 2**

**Case 3**

**Case 5**
