# Supplementary material for: Myeloblasts transition to megakaryoblastic immunophenotypes over time in some patients with myelodysplastic syndromes
Source: PLoS One. 2023 Sep 20;18(9):e0291662. doi: 10.1371/journal.pone.0291662 (PMC10511088; doi:10.1371/journal.pone.0291662)
Supplement: S4 Fig — [Left panel] A cytospin slide prepared from the FCM sample showing that platelets adhered to various cells (arrows). [Middle panel] A: Singlet events were displayed on the FSC versus SSC plot, and cells with low SSC were gated. A P1 gate was used to identify platelets. B: Low SSC cells (gated in panel A) were displayed on the CD34 versus CD45 plot. CD34+ blasts (red dots) and CD45-bright cells were gated. C: CD45-bright cells in Panel B were displayed on SSC versus CD45 plots, and lymphocytes (blue dots) were gated. D: Events in P1 (Panel A) were displayed on SSC versus CD41 plots and platelets (CD41+ cells) were gated. E: CD34+ blasts were displayed on the CD41 versus CD33 plot. CD41+ blasts (BL41+) and CD41- blasts (BL41-) were gated. F: Lymphocytes were displayed on the CD41 versus CD33 plot. CD41+ lymphocytes (Ly41+) and CD41- lymphocytes (Ly41-) were gated. [Right panel] Images of various cell fractions gated in the middle panel. A: Platelets. B: CD41+ blasts. C: CD41- blasts. D: CD41+ lymphocytes. E: CD41- lymphocytes. The arrow head indicate RBC ghost, which is observed in every cell fraction. The arrows indicate platelets adhering to leukocytes, which caused false CD41-positivity in leukocytes. Platelet adherence was observed in CD41-positive cell fractions (B and D), but not in CD41-negative cell fractions (C and E). Note that because the imaging FCM captures cell images from one direction, platelet adherence to the back surface of leukocytes was not detected. (DOCX) [file pone.0291662.s004.docx]

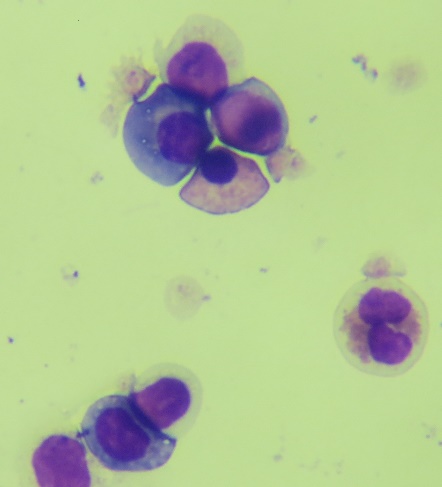


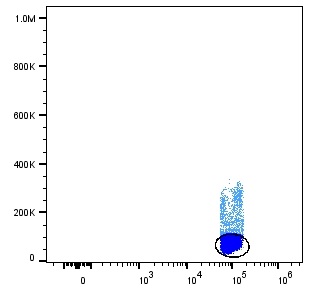

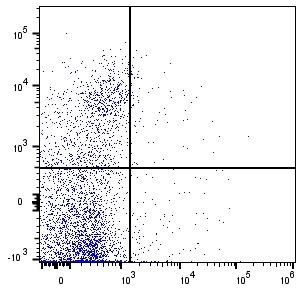


SSC

CD45

CD41

CD33

Ly41+

Ly41―

**(C)**

**(F)**


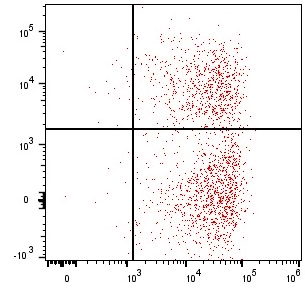

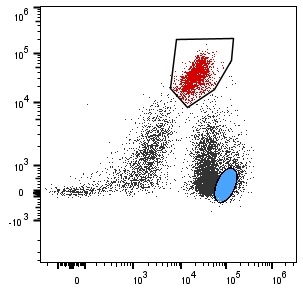


CD34

CD45

CD41

CD33

BL41+

BL41-

**(B)**

**(E)**


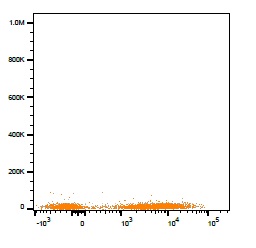

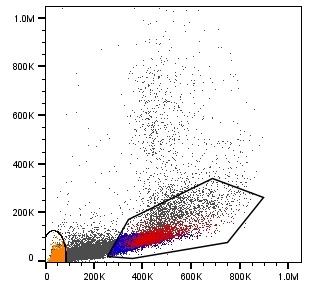


SSC

FSC

SSC

CD41

Platelets

**(A)**

**(D)**


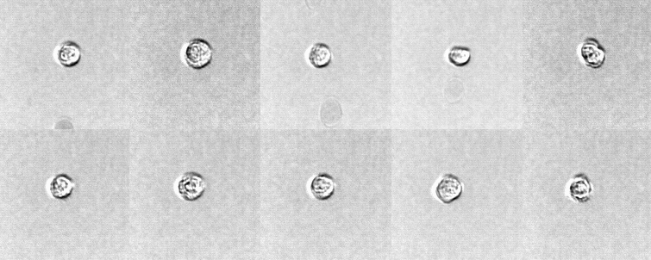


**(E)**


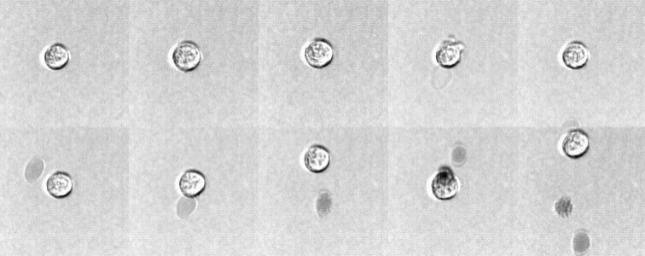


**(C)**


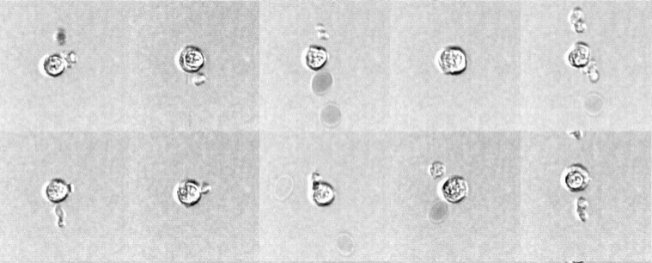


**(D)**


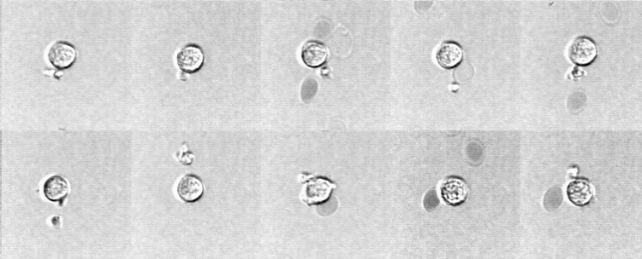


**(B)**


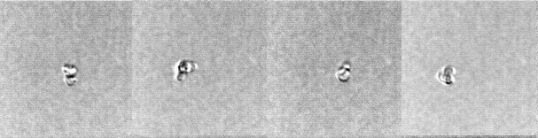


**(A)**

**Supplementary Figure 4A. A CD41 false-positive case**

P1
