# Supplementary material for: Myeloblasts transition to megakaryoblastic immunophenotypes over time in some patients with myelodysplastic syndromes
Source: PLoS One. 2023 Sep 20;18(9):e0291662. doi: 10.1371/journal.pone.0291662 (PMC10511088; doi:10.1371/journal.pone.0291662)
Supplement: Attachment — Submitted filename: Response to Reviewers.docx [file pone.0291662.s008.docx]

Dear Dr. Thomas,

Thank you for reviewing and commenting on our manuscript titled “Myeloblasts transition to megakaryoblastic immunophenotypes over time in some patients with myelodysplastic syndromes.” We appreciate your valuable time and efforts.

Here, I am resubmitting our manuscript, modified according to the comments from the editor and reviewer as well as the style requirements of PLOS ONE. The modified parts are highlighted in red in the revised manuscript.

The original comments from the editor and reviewer and our responses to them are presented below in a point-by-point manner.

We hope that the revised manuscript is now acceptable for publication in PLOS ONE.

Thank you for your consideration. I look forward to hearing from you.

Kiyoyuki Ogata, MD

***Responses to the editor***

**The editor’s comment 1**

What proportion of CD41+ vs CD41- negative patients were actually t-MN, given the complex karyotypes noted. Should be discussed if no data.

**Our response**

Ten patients in our cohort had therapy-related myeloid neoplasms (t-MN), which was unrelated to their CD41-positivity. In this revision, we showed these data in Table 1 and S2 Table.

**The editor’s comment 2**

Can we do a correlation plot of CD41 vs CD61 in the few patients tested and CD41 vs CD42b? Can we draw conclusions on what added benefit CD61 vs CD42b would have in the flow diagnostic panel?

**Our response**

We examined the expression of CD41, CD42b, and CD61 on blasts in the same tube using flow cytometry; this was respectively done for five CD41+ patients, as described in the Methods and Table 2. In all five patients, the expression level of CD41 and CD61 on blasts showed a very strong correlation as shown in Figure 3 (Case 6). In the revised manuscript, we have added the plot of CD41 *vs*. CD61 for the other four cases (S2 Fig). Such a strong correlation between CD41 and CD61 expression is also observed in normal platelets (Ann Lab Med. 2014. 34(6): 471–474), as CD41 and CD61 molecules are known to form a gpIIb/IIIa complex.

Further, in our five cases, addition of the CD61 *vs*. CD42b plot had no benefit in diagnosing patients with CD41+ MDS. However, because the number of cases was small, we avoided drawing a conclusion regarding the diagnostic value of adding CD61 (or the CD61 *vs*. CD42b plot); instead, we showed the CD41 *vs*. CD61 plots from all five cases as described above and added a comment on this issue (pages 14 and 18).

As different laboratories are using different antibody panels in diagnostic flow cytometry, the data of CD61 expression in addition to those of CD41 and CD42b shown in this manuscript can be helpful to some laboratories.

**The editor’s comment 3**

Was CD110, TpoR, also expressed in any of the CD41+ blasts?

**Our response**

This is an important issue from various viewpoints, including the positive or negative effect of TpoR agonists on patients with CD41+ MDS. Unfortunately, we have not yet examined TpoR expression in any of the cases.

**The editor’s comment 4**

It would be desirable to present CD41+ (at any time) vs CD41- patients in a side by side table with P-values of significance - easy for the reader to see the differences.

**Our response**

In this revision, we created a new table (S2 Table), wherein basic characteristics were compared between CD41+ and CD41- patients in a side-by-side fashion with P-values.

**The editor’s comment 5**

Early platelet recovery is a sign of azacitidine response - can we comment on the duration of azacitidine (+/- ven) for any of the CD41+ vs CD41- negative patients?

**Our response**

In this revision, we compared the number of azacitidine (+/- ven) cycles administered between the CD41+ and CD41- patients. More specifically, the number of cycles until the detection of CD41-positivity in former patients and that until the last flow cytometry analysis in latter patients were compared. However, we did not find a significant difference in the number (S2 Table).

***Responses to the reviewer***

Thank you very much for reviewing and commenting on our manuscript, which helped improve our manuscript. We highly appreciate your valuable time.

**The reviewer’s major comment**

The way that this study was undertaken introduces substantial bias. The patients were eligible for HMA therapy, so enriched for high risk. Serial bone marrow aspirates were performed only for poor responders and not systematically for all patients. This means that we simply don’t know whether CD41 expression might also arise in lower risk patients or those apparently responding to HMA. This should be acknowledged as a confounding factor.

**Our response**

We completely agree with this comment. We have acknowledged this important point in the discussion of the revised manuscript (page 19-20).

**The reviewer’s minor comment**

Some of the references are incomplete (online prepublication details only).

**Our response**

We have examined and corrected the references thoroughly.
